# Two Strategies for the Dosage of Acenocoumarol Co-Administered with Rifampicin in Staphylococcal Prosthetic Valve Endocarditis

**DOI:** 10.3390/antibiotics10010038

**Published:** 2021-01-03

**Authors:** Marcin Wełnicki, Małgorzata Buksińska-Lisik, Artur Mamcarz

**Affiliations:** 3rd Department of Internal Medicine and Cardiology, Medical University of Warsaw, Bursztynowa 2, 04-479 Warsaw, Poland; mblisik@wp.pl (M.B.-L.); a.mamcarz@3med.pl (A.M.)

**Keywords:** drug interactions, endocarditis, rifampicin, vitamin K antagonists

## Abstract

According to current European Society of Cardiology guidelines, for staphylococcal prosthetic valve endocarditis, rifampicin should be one of the drugs used. However, there is a concomitant need for vitamin K antagonists in patients with mechanical prostheses. It is widely known that rifampicin interacts with vitamin K antagonists (VKA), and this interaction makes it difficult to maintain the INR (international normalized ratio) value in the therapeutic range. We present two clinical cases of staphylococcal prosthetic valve endocarditis patients. Two different strategies for dealing with adverse drug interactions have been applied. In the first case, the dose of warfarin was up-titrated until the optimal INR value was obtained. In the second case, due to the history of labile INR values, a decision was made to modify the dosage of warfarin, taking into account pharmacological aspects of drug interactions.

## 1. Introduction

The time of vitamin K antagonists (VKA) seems to pass slowly. Once commonly used acenocoumarol or warfarin are being replaced by factor Xa inhibitors (apixaban, riwaroxaban) or direct thrombin inhibitor (dabigatran). This is clearly visible, for example, in the following guidelines for the management of patients with atrial fibrillation or pulmonary embolism. In subsequent documents, the recommendations on non-vitamin K oral anticoagulants (NOACs) increase in strength [1,2,3,4]. However, the prosthetic heart valves replacement remains a leading indication for VKA [5]. One of the main disadvantages associated with VKA is the narrow therapeutic index and the large individual variability of the dose–effect relationship [6]. Obtaining a stable and therapeutic INR (international normalized ratio) is often difficult. Insufficient anticoagulation same as VKA overdosing (determined by too low or too high INR value respectively) can result in serious adverse events. The cause of thrombus formation on a prosthetic valve is not only in non-compliance with regard to the use of VKA in general, but also the maintenance of sub-therapeutic INR values [7,8,9,10]. At the same time, the lability of the INR value is one of the risk factors for severe haemorrhages according to the HAS-BLEED scale [11]. A breakthrough scale developed for patients with atrial fibrillation (AF) without major valvular events also turned out to be useful in cases with AF and concomitant valvular heart disease (VHD) [12]. Large dose response variations on VKA are influenced most of all by pharmacokinetic aspects of the drug. These in turn partly depend on patient’s genetic profile, while in many cases drug interactions play a key role. The interaction between warfarin and rifampicin is well known. If both drugs are administered at the same time, it is much harder to establish therapeutic INR, as rifampicin reduces the effect of warfarin. To understand the nature of this relationship, however, we need to analyze the influence of both substances on the pharmacokinetic properties. Rifampicin is a potent p450 cytochrome inducer. In the case of warfarin, and more specifically its more potent S-enantiomer, a key role in metabolism is played by cytochrome P450 (CYP) 2C9. Acenocumarol and phenprocoumon (rarely used VKA) elimination also depends on the activity of 2C9 however, its impact is smaller, the smallest in the case of phenprocoumon [6]. Moreover, it has been shown that rifampicin accelerates the process of warfarin elimination by reducing its half-life time from about 47 to 18 h. No effect on maximal serum concertation o warfarin [6,13,14] is being observed. We are not aware of similar data as far as acenocumarol is concerned and we expect that the effect of rifampicin has a similar mechanism. The half-life of acenocoumarol is 8–11 h, the maximum serum concentration occurs 1–3 h after oral administration. The VKA characteristics of the medicinal product assume their once-daily dosing and dose modification depending on the INR value [6,13,14]. Thus, the knowledge of the mechanisms of drug interaction may justify the attempt to individualize the VKA dosing regimen. We present descriptions of two clinical cases in which, due to staphylococcal prosthetic valve endocarditis, it was necessary to use acenocoumarol and rifampicin simultaneously. In both cases, the patients signed a written consent to use their medical data for scientific purposes, including the publication of a description of clinical cases.

## 2. Case Reports

### 2.1. Case 1

51-year-old man with Marfan syndrome, after mitral valve repair due to its regurgitation in 2000, after Bentall procedure (St. Jude Medical mechanical valve) and concomitant coronary artery bypass grafting (single SV/RCA graft) in 2015, with chronic heart failure and permanent atrial fibrillation was admitted to the Clinic in March 2017 due to feverish conditions. The echocardiography showed vegetation on the aortic prosthesis, and blood culture revealed the presence of methicillin-susceptible *Staphylococcus aureus* (MSSA).

**Figure 1:**
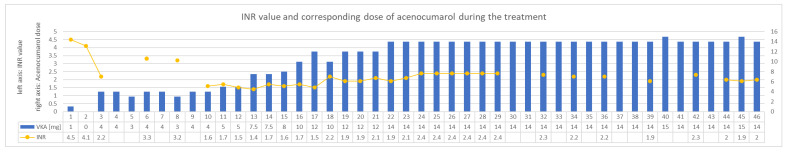
INR values and treatment in following days of concomitant administration of acenocumarol and rifampicin—case 1. INR—international normalized ratio. VKA—vitamin K antagonist.

The treatment was implemented in accordance with the guidelines as follows: cloxacillin, gentamicin, rifampicin [15,16,17]. During antibiotic therapy, there were difficulties in maintaining therapeutic INR values. Because of non-therapeutic INR, the patient required concomitant use of low molecular weight heparin (LMWH) for several consecutive days. Eventually stable and almost therapeutic INR was reached with 14 mg of acenocoumarol administered once daily. Changes in the dosage of acenocoumarol and corresponding INR values on subsequent days of antibiotic therapy are presented in Figure 1. As for the other blood test results—creatinine concentration remained within the normal range throughout the hospitalization (57–66 μmol/L (normal range 45–84 μmol/L); GFR about 120 mL/min/1.73 m^2^). The haemoglibin concentration was also stable at about 13 g/dl. No thrombocytopenia was observed. Transient increases in the activity of transaminases (ALT up to 266 U/L (normal range < 35 U/L) and AST up to 132 U/L (normal range < 35 U/L)) were observed, but these parameters normalized towards the end of the treatment.

### 2.2. Case 2

Prosthetic valve endocarditis was diagnosed in 72-year-old woman presented with chronic heart failure, permanent atrial fibrillation and a history of multiple heart valve interventions. She had a background of mitral commissurotomy (years ago) and surgical valves replacement in 2000 (a mechanical prosthesis in the mitral position and bioprosthesis in the tricuspid position and replaced later in 2011). Moreover, she underwent a pacemaker implantation in 2000 and combined aortic valve disease with the predominant stenosis diagnosed in mean time.

**Figure 2:**
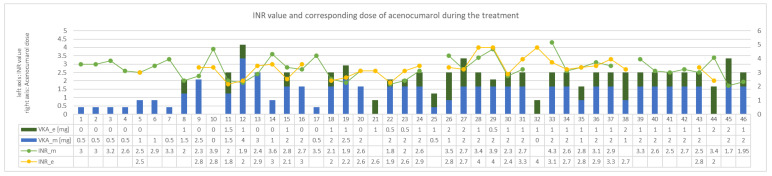
INR values and treatment in following days of concomitant administration of acenocumarol and rifampicin—case 2. VKA_e—dose of the vitamin K antagonist given in the evening. VKA_m dose of the vitamin K antagonist given in the morning. INR_m—INR value in the morning, INR_e—INR value in the evening. INR—international normalized ratio. VKA—vitamin K antagonist.

MSSA was grown in blood cultures so cloxacillin, gentamicin and rifampicin were implemented in accordance with the ESC guidelines [15,16,17]. In the past, most likely, due to the coexistence of hepatic cirrhosis secondary to right heart failure, INR values above 3.5 were often found. Therefore, the patient had her own portable INR measuring device. To date, low doses of acenocoumarol (approximately 0.5 mg daily) have been used in chronic treatment. Anticipating difficulties in the dosage of VKA with concomitant use of rifampicin, it was decided to increase the frequency of INR measurements to two per day and the possibility of using low doses of acenocoumarol twice a day. In the following days, the INR was maintained within the therapeutic range using this strategy, despite the intake of rifampicin. Changes in the dosage of acenocoumarol along corresponding INR values on subsequent days of antibiotic therapy are presented in Figure 2. As for the other blood test results, creatinine concentration was slightly alleviated at the beginning of the treatment (86 μmol/L) and normalized toward its end (75 μmol/L, GFR > 60 mL/min/1.73 m^2^). The concentration of haemoglibin was stable and amounted to about 9–10 g/dl (patient initially with anemia of chronic diseases). No thrombocytopenia was observed. Transient increases in the activity of transaminases were also observed, as in the first case, but were rather slight (ALT up to 46 U/L and AST up to 59 U/L).

## 3. Discussion

In the first described case, the standard rule of dosing VKA once a day and up-titration of the dose of the drug until the target reached INR values were adopted. A stable INR value was obtained only at a dose of 14 mg acenocoumarol per day. Over the entire analyzed period, the patient took an average of 10.5 mg (SD ± 4.7) of acenocoumarol per day. Before starting antibiotic therapy, the average daily dose of acenocoumarol was 4.3 mg (last seven days, moving average). During the rifampicin treatment period, the demand for acenocoumarol increased approximately 2.4-fold. The average INR in the analyzed period was 2.2 (SD ± 0.7). It should be noted that during the non-therapeutic INR period, the patient was administered a therapeutic dose of LMWH twice a day. In addition, the stable INR values were subtherapeutic.

Sharif Khan et al. showed that sub-therapeutic INR values may be responsible for nearly half of the thrombus formation in patients with prosthetic heart valves [18]. More recently, Thorne et al. indicated that in the case of coexistence of AF in patients with prosthetic valves, the target INR should be closer to 3.0 [10]. In the described case, however, both the doses of acenocoumarol used and the INR values seemed optimal in the situation. In the clinical case described by Mitzer et al., the authors also adopted a similar strategy: using heparin bridging therapy [19] in the period of non-therapeutic INR. The use of subcutaneous LMWH could be an acceptable alternative to unfractionated heparin (UFH) for bridging [20]. Moreover, in the presented case, such protocol was found to be a convenient and effective method, especially for single, nonconsecutive days.

Considering the mechanism of interaction between rifampicin and warfarin, however, one may be concerned whether the high one-daily dose of the drug does not increase its maximum concentration in the serum above acceptable toxic level. These could, especially for patients with pre-existing labile INR values, result in increased risk of haemorrhagic complications. For this reason, in the second described case a strategy of increased INR control frequency was undertaken. In the event of a significant reduction in INR in the evening compared to the morning hours, the patient received an additional dose of acenocoumarol. Under normal conditions, increase INR control frequency requires more daily blood collection. In this case, however, the patient agreed to monitor the INR value using her own portable device. In the course of treatment of endocarditis and in the conditions of complex antibiotic therapy the average daily dose of acenocumarol in second case was 2.3 mg (SD ± 1.1) per day. Before the hospitalization due to endocarditis, patient took on average 0.5 mg of acenocumarol per day (last seven days, moving average), so the demand for drug increased 4.6-fold. The average INR obtained in the analyzed period was 2.7 (SD ± 0.6). It is noteworthy that 72% of INR measurements remained above 2.5. Obtaining over 70% of measurements in the therapeutic field is a very good treatment effect. Even in the case of patients with AF without a prosthetic valve, in such a situation, one might wonder about the merits of replacing the VKA with one of the NOACs [1]. On the other hand, 30% of the measurements indicated INR values equal or above 3.0.

It is also worth paying attention to the differences between the relative increase in the demand for acenocoumarol. In the first case, the average dose of the patient during the rifampicin treatment period was 2.4 times higher than before the treatment. In the second case, the average dose of the drug was 4.6 times higher than in the period before antibiotic therapy. Therefore, it seems that the quantitative effect of the interaction of rifampicin and acenocoumarol may also be influenced by the genetically determined cytochrome [13,14]. Therefore, the increase of the frequency of INR controls and to individualize the dosage of the anticoagulant, might be needed.

Finally, it is worth commenting why the use of rifampicin was found to be the cause of INR lability. The treatment regimen of chronic diseases in the presented patients did not change significantly. The patients remained stable in terms of cardiovascular parameters throughout the hospitalization period. In both cases, the VKAs were used before and the INR values were well monitored and controlled. Therefore, only the antibiotics used could potentially have an influence on the INR lability. In both cases, both the pathogen and the treatment regimen used were the same. As far as antibiotics are concerned cloxacillin, gentamicin and rifampicin were administered. Such selection of antibiotics is consistent with both the current ESC guidelines and the opinion of many experts. It is worth emphasizing though, that management of endocarditis is still controversial, and both diagnostic and therapeutic decisions have to be made by taking into account many individual variables [15,17,21,22,23]. Among the antibiotics used, the aminoglycoside (gentamicin) does not affect the effectiveness of VKA, while the penicillin derivative (cloxacillin), may impact the effect of VKA. Several past clinical cases were described in which the concomitant use of cloxacillin and warfarin made it difficult to obtain therapeutic INR. They did not explain, however, the mechanism of such interaction and it cannot rule out influence of other drugs used [24]. It is commonly believed that the concomitant use of cloxacillin and VKA may enhance the effect of this group of drugs and increase the risk of bleeding [25]. However, in our cases the strongest interaction occurs with rifampicin, which reduces the effectiveness of VKA by the mechanism described in the introduction [6,14,26].

When commenting on the described cases, it is also worth referring to several recently published studies. Ma et al. performed a meta-analysis of adjunctive rifampicin usage for the treatment of *Staphylococcus aureus* bacteraemia [27]. The authors point out that there is no clear evidence of lower mortality rate in patients who received rifampicin as a part of multi-drug regimen. However, this analysis suffered from a high heterogeneity of the results due to the variety of infection locations (pneumonia, osteitis, endocarditis with and without artificial valves, etc.). A subgroup analysis showed that in MSSA infections, such as in the clinical cases described above, rifampicin may, however, improve prognosis [27]. Another clinical aspect that must not be forgotten is the risk of acute kidney injury (AKI) in patients with endocarditis. According to Ortiz-Soriano et al., AKI may occur in up to two out of three patients with endocarditis [28]. Kidney function is a key variable in dose titration of many antibiotics, but not with rifampicin. It should be noted that in both cases described, the renal parameters were stable during treatment.

In the end it needs to be highlighted that in both analyzed cases, there were no haemorrhagic nor thromboembolic complications, so the treatment strategy was both effective and safe. It is worth noting, however, that a few months after the described hospitalization due to endocarditis, the patient described as case 2 was re-admitted to the Department. The reason for re-admission to the hospital was anemization in the course of massive epistaxis. It should be emphasized that the patient was taking VKA in the standard, original dosing regimen at the time of the onset of the epistaxis.

## 4. Conclusions

At the moment, there is no single recommended strategy for dealing with the adverse interaction of rifampicin with VKA. In most cases, the once-daily dosing schedule is followed, and the dose escalates until a therapeutic INR is obtained. In cases on non-therapeutic INR low molecular weight heparin is often used simultaneously with VKA. However, in patients at higher risk of bleeding complications, a different strategy could be considered. Considering the mechanism of pharmacokinetic interaction between rifampicin and VKA, dosing of the anticoagulant twice daily with increased control INR measurements may be both effective and safe.

## Data Availability

Data is contained within the article.
